# Multifragmentary Thoracic Fracture as an Initial Sign of Solitary Bone Plasmacytoma: Trauma-Oncologic Approach in a Mexican Patient

**DOI:** 10.7759/cureus.94364

**Published:** 2025-10-11

**Authors:** Irving A Buenfil-Cruz, Oscar A Gonzalez-Martinez, Victor M Ayuso-Diaz, Rene E Gamboa-Garcia, Adriana F Arrieta-Martin, Angelica Moreno-Enriquez

**Affiliations:** 1 Department of Orthopaedic Surgery and Traumatology, Hospital Regional Elvia Carrillo Puerto, Institute for Social Security and Services for State Workers (ISSSTE), Yucatan, MEX; 2 Faculty of Medicine, Autonomous University of Yucatán (UADY), Yucatan, MEX; 3 Research and Education Division, Medical Care and Research, Yucatan, MEX; 4 Genomic-Metabolic Unit, University Marista of Merida, Yucatan, MEX; 5 Metabolic Genomic Unit, Marista University of Merida, Yucatán, MEX

**Keywords:** cd38, lambda light chains, pathological vertebral fracture, solitary bone plasmacytoma, spine oncology, thoracic spine fracture

## Abstract

Although multifragmentary thoracic fractures are usually associated with high-energy trauma, they may also be the initial manifestation of an underlying neoplasm. Although infrequent, Solitary Bone Plasmacytoma (SBP) can present with severe vertebral collapse even after minimal trauma, highlighting the importance of considering oncological aetiologies in patients with atypical vertebral pain or slow progression. We present the case of a 48-year-old Mexican patient with a multifragmentary T7 fracture secondary to dorsal trauma, which was initially managed conservatively. The appearance of progressive neurological deficit and structural instability prompted advanced imaging studies. Magnetic resonance imaging (MRI) revealed 90% collapse of the T7 vertebral body with retropulsion of the posterior wall, and positron emission tomography/computed tomography (PET/CT) showed focal hypermetabolic uptake at T7/T8 compatible with a tumour lesion and spinal cord compression. Decompression was performed via laminectomy and transpedicular fixation, with resection of the abnormal tissue for histopathological and immunohistochemical analysis. The study confirmed SBP with a CD38⁺ immunophenotype and restriction to lambda light chains. Multidisciplinary management included surgery, fractionated radiotherapy (45 Gy), zoledronic acid, lenalidomide, and denosumab. The patient experienced favourable clinical evolution and partial neurological recovery (strength 5/5 in the lower limbs and no residual sensory deficit).

## Introduction

Multifragmentary thoracic vertebral fractures, although infrequent, represent a clinical challenge due to their potential structural instability and associated neurological risk. Notwithstanding the prevalence of high-energy trauma as their most common origin - including, but not limited to, vehicular accidents, falls from heights and sports injuries - there are scenarios in which the magnitude or pattern does not correlate with the aforementioned mechanism. Consequently, consideration must be given to underlying etiologies [1, 2, 3]. These include primary or secondary vertebral neoplasms, among which solitary bone plasmacytoma (SBP) is notable for its low frequency but high clinical impact [4, 5, 6].

SBP is a neoplasm of malignant origin, characterised by the presence of a solitary bone lesion without evidence of bone marrow involvement or systemic metastases. It accounts for approximately 5% of monoclonal gammopathies, with an estimated overall incidence of 0.15 to 0.30 per 100,000 population [5, 6, 7]. The spine, notably the mid-thoracic region, is a prevalent site of manifestation [3, 4, 5]. In such cases, local tumour infiltration profoundly alters bone architecture through cytokine-mediated mechanisms (IL‑6, receptor activator of nuclear factor kappa-Β ligand (RANKL)) and activation of intracellular pathways such as nuclear factor kappa-light-chain-enhancer of activated B cells (NF‑κB), favouring vertebral collapse even in the absence of significant trauma [7, 8, 9].

As evidenced by numerous case reports, the initial presentation of SBP was often a pathological fracture, which was initially misdiagnosed as an isolated traumatic injury [9, 10]. As early diagnosis can modify the functional and oncological prognosis, it is essential to maintain a high index of suspicion in patients with atypical vertebral fractures, neurological progression or disproportionate pain. The evaluation process should encompass imaging studies, such as magnetic resonance imaging (MRI) and positron emission tomography/computed tomography (PET/CT), in conjunction with histopathological and immunohistochemical analysis (CD38+, kappa or lambda light chains) to substantiate the diagnosis [10, 11].

From a genetic perspective, recurrent alterations have been identified in genes such as *TRAF3*, *CYLD*, *BIRC3 *and *MAP3K14*, which are associated with dysfunction in plasma cell regulation and risk of progression to multiple myeloma [1, 2, 5, 10]. These molecular features allow for enhanced risk stratification and therapeutic selection.

The treatment of vertebral SBP with neurological involvement involves surgical decompression and transpedicular fixation, followed by conformal radiotherapy (typically 40 to 50 Gy) as recommended in current guidelines [6]. In patients with a high risk of progression or extensive bone involvement, systemic therapies such as lenalidomide, zoledronic acid or denosumab are administered [6, 7]. The utilisation of validated clinical tools such as the Spinal Instability Neoplastic Score (SINS) is imperative in determining the necessity for surgical intervention in such cases [10, 12, 13].

## Case presentation

A 48-year-old male patient, who had been in good health until then, presented at the emergency department following a direct dorsal trauma resulting from an act of interpersonal violence. Upon admission, the patient was found to be hemodynamically stable, ambulatory without assistance, and reported experiencing pain in the dorsal region. A physical examination was conducted, which revealed the presence of pain upon palpation in the thoracic midline. In addition, functional limitations were identified, specifically a reduction in trunk flexion and extension due to localized pain and paravertebral muscle spasm, and no evidence of neurological deficit was observed (classified as ASIA E, according to the American Spinal Injury Association (ASIA) impairment scale). A radiographic (X-ray) evaluation revealed a multifragmentary fracture at T7, accompanied by a 10% loss of vertebral height without evidence of displacement. The classification was determined to be Arbeitsgemeinschaft für Osteosynthesefragen (AO) Spine Type A4, Thoracolumbar Injury Classification and Severity Score (TLICS) 2, and Spinal Instability Neoplastic Score (SINS) 6, indicating the necessity for conservative management.

**Figure 1:**
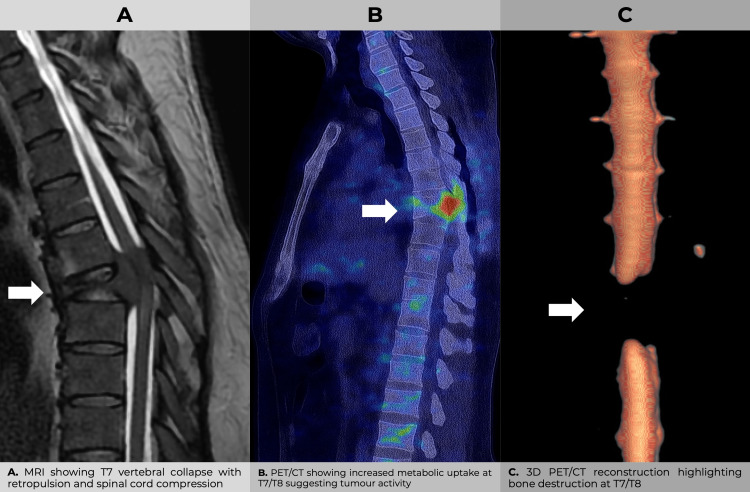
Multimodal imaging of the thoracic spine in a patient with T7 vertebral involvement. Sagittal MRI, PET/CT and 3D reconstructions show structural collapse, metabolic activity and bone destruction at the T7/T8 level. These findings collectively support tumour-related vertebral damage with spinal cord compromise.

Over the course of the initial four-week period, the patient demonstrated a degree of improvement. However, following mild exertion, the patient reported acute dorsal pain, weakness in the pelvic limbs and difficulty in standing. Subsequent re-evaluation resulted in a rating of 3/5 for strength in the lower limbs, alongside documented preserved sensitivity, thus prompting further imaging studies. Magnetic resonance imaging (MRI) revealed a 90% collapse of the T7 vertebral body, accompanied by retropulsion towards the spinal canal. Additionally, the T2-weighted image demonstrated an intense signal, indicating potential tumour infiltration (Figure 1A). The lesion was reclassified as AO A4N, TLICS 7, SINS 11. A PET/CT scan revealed focal hypermetabolic uptake in T7 and T8, indicative of tumour activity (Figure 1B). Three-dimensional reconstruction confirmed the presence of thoracic structural involvement (Figure 1C). 

In view of the suspicion of neoplasia, extension studies were requested. Serum tumor markers (alpha-fetoprotein (AFP), carcinoembryonic antigen (CEA), and carbohydrate antigen 19-9 (CA 19-9)) were found to be within normal parameters. However, protein electrophoresis revealed a monoclonal M spike, and immunofixation confirmed lambda-type IgG (Table 1).

**Table 1:** Report summarises the results of tumour markers and complementary studies.

Test	Result	Reference Range	Interpretation
Alpha-fetoprotein (AFP)	3.2 ng/mL	0.0 - 8.0 ng/mL	Normal
Carcinoembryonic Antigen (CEA)	1.5 ng/mL	0.0 - 5.0 ng/mL	Normal
Carbohydrage Antigen (CA) 19-9	12.0 U/mL	0 - 37 U/mL	Normal
Serum Protein Electrophoresis	Monoclonal band	Not detectable	Abnormal (M-protein spike detected)
Serum Immunofixation	Monoclonal IgG Lambda	Not detectable	Abnormal (monoclonal gammopathy identified)

**Figure 2:**
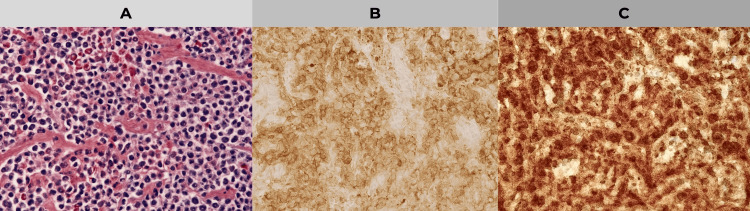
Histopathological and immunohistochemical study of T7 vertebral biopsy. A: Histology image with hematoxylin-eosin (HE) staining, showing an infiltration of atypical plasma cells in the bone tissue, characteristic of a plasmacytoma. B: Immunohistochemistry with CD38 marker, showing positivity in plasma cells, confirming their origin and abnormal proliferation. C: Immunohistochemistry with lambda light chains, showing clonal restriction of these chains, a finding consistent with plasma cell neoplasia.

In view of the progressive neurological deficit, the indication was for urgent surgical intervention involving spinal cord decompression and transpedicular fixation. The following screws were placed: T5, T6, T8 and T9. In addition, a T6-T7 laminectomy with biopsy was performed. During the intervention, friable tissue was identified adhering to the dural sac. Histopathological analysis revealed the presence of atypical plasma cells, and immunohistochemistry confirmed CD38 expression and clonal restriction to lambda light chains (Figure 2). 

Multidisciplinary treatment was initiated at postoperative week 2 with monthly intravenous administration of zoledronic acid at a dose of 4 mg per cycle. In the third week of treatment, 3D conformal radiotherapy was initiated, with a total dose of 45 Gy administered in 25 fractions (1.8 Gy/fraction, five weekly sessions). In the week following the completion of radiotherapy (week 9), the patient commenced chemotherapy with lenalidomide (25 mg/day for 21 consecutive days, repeated every 28-day cycle) and denosumab 120 mg subcutaneously every 4 weeks.

During the treatment period, the patient was evaluated using the Common Terminology Criteria for Adverse Events (CTCAE) v5.0 scale, which reported only grade 1 cutaneous toxicity and grade 1 fatigue, with no major adverse effects. No surgical complications of Clavien-Dindo I classification were observed. The biochemical follow-up included inflammatory markers, which demonstrated a progressive decrease and clinical concordance (Table 2). 

**Table 2:** The evolution of systemic inflammation markers during postoperative follow-up. CRP: C-reactive protein; ESR: erythrocyte sedimentation rate

Day	CRP (mg/L)	ESR (mm/h)	Leukocytes (x10³/μL)
Admission	18.2	45	12.8
Day 3	10.5	35	10.4
Day 7	5.4	28	9.2
Day 14	2.1	18	7.6
Day 30	0.6	10	6.8
Reference Range	< 5 mg/L	< 20 mm/h	4.0 – 10.0 ×10³/μL

Three months following the implementation of the multidisciplinary approach, the patient was able to walk without assistance, exhibiting a complete recovery of muscle strength (5/5) and no pain or neurological dysfunction. A recent PET/CT scan revealed local metabolic remission and the absence of active systemic disease. At present, the patient is reported to be in a stable clinical condition, with no sensory alterations or signs of progression. The multifaceted intervention, executed with the collaborative involvement of the following specialties - traumatology, spine surgery, radiology, pathology, radiotherapy and haematology - facilitated not only a prompt diagnosis but also an efficacious treatment in this atypical case of multifragmentary thoracic fracture secondary to a solitary bone plasmacytoma. The clinical evolution, in conjunction with the imaging and laboratory findings, corroborates the efficacy of the interdisciplinary approach, thereby attaining a highly favourable functional and oncological prognosis.

## Discussion

Solitary bone plasmacytoma (SBP) is an uncommon but clinically significant form of plasma cell neoplasm. The diagnosis of this condition may be delayed when it presents as an apparently traumatic vertebral fracture, as was the case in this instance. Thoracic multifragmentary fractures are typically attributed to high-energy mechanisms; nevertheless, the presence of disproportionate symptoms, neurological progression or poor evolution under conservative management compels the search for underlying causes, including haematologic pathologies [1, 2, 13, 14, 15].

The extant literature has documented cases in which the presence of a plasma cell myeloma (PM) manifests as an unstable fracture with progressive vertebral body collapse and spinal cord compression, in the absence of systemic criteria for multiple myeloma [3,4,5,16]. This clinical pattern suggests structural fragility induced by tumour infiltration, mediated by cytokines such as IL-6 and NF-κB activation mechanisms [4]. In this case, the utilisation of structured classifications (AO Spine, TLICS, ASIA and SINS) facilitated therapeutic decision-making, aligning with international standards [5,17,18].

The surgical indication was evident in the presence of neurological deficit and signs of instability. The intervention facilitated diagnostic resection and therapeutic decompression, in addition to mechanical stabilisation. The efficacy of this approach is substantiated by studies demonstrating that surgical intervention in SBP** **with neurological compromise results in a substantial enhancement in functional prognosis [6,19].

Consequently, an oncological therapeutic scheme was formulated in accordance with international guidelines, encompassing the delivery of 45 Gy of conformal radiotherapy in 25 fractions, in conjunction with zoledronic acid, lenalidomide, and denosumab, to forestall skeletal-related events. The selection of these agents was informed by their documented effectiveness in delaying progression to multiple myeloma and reducing recurrences [13,14,15]. It is important to acknowledge the reviewer's observation that the sequence of treatments was meticulously planned both chronologically and clinically. The treatment plan commenced with surgery (week 0), followed by zoledronic acid (week 2), radiotherapy (weeks 3-7), and subsequently systemic treatment (week 9 onwards).

During subsequent follow-up, the patient was evaluated using the CTCAE v5.0 scale for oncologic toxicity, and no major adverse events were observed. No surgical complications were observed according to the Clavien-Dindo classification. Therapeutic response was monitored by means of inflammatory markers (C-reactive protein (CRP), erythrocyte sedimentation rate (ESR), leukocytes), sequential imaging and neurological function.

## Conclusions

A multifragmentary thoracic fracture with atypical evolution is a clear indication of a bone neoplasm, such as solitary bone plasmacytoma. The absence of multiple myeloma criteria and the identification of an IgG lambda monoclonal gammopathy allowed us to confirm the diagnosis. The integration of advanced imaging, immunohistochemical studies and a multidisciplinary approach was essential for a timely and effective intervention. This case shows that oncologic causes must be considered in unusual vertebral fractures. Close surveillance is vital to detect clonal progression and optimise functional and oncologic prognosis.
